# Bone Mesenchymal Stem Cell-Derived sEV-Encapsulated Thermosensitive Hydrogels Accelerate Osteogenesis and Angiogenesis by Release of Exosomal miR-21

**DOI:** 10.3389/fbioe.2021.829136

**Published:** 2022-01-19

**Authors:** Di Wu, Hao Qin, Zixuan Wang, Mingzhao Yu, Zhe Liu, Hao Peng, Leilei Liang, Changqing Zhang, Xiaojuan Wei

**Affiliations:** ^1^ Department of Orthopedic Surgery, Shanghai Jiao Tong University Affiliated Shanghai Sixth People’s Hospital, Shanghai, China; ^2^ Center for Reproductive Medicine, Department of Obstetrics and Gynecology, Peking University Third Hospital, Beijing, China; ^3^ Department of Mechanical Engineering, Tsinghua University, Beijing, China; ^4^ National Cancer Center, Chinese Academy of Medical Sciences and Peking Union Medical College, Beijing, China; ^5^ Institute of Microsurgery on Extremities, Shanghai Jiao Tong University Affiliated, Shanghai Sixth People’s Hospital, Shanghai, China

**Keywords:** thermosensitive hydrogel, small extracellular vesicle, release, bone regeneration, angiogenesis

## Abstract

Angiogenesis has been recognized to play an essential role in remodeling new bone (osteogenesis). Small extracellular vesicles (sEVs), the endogenously secreted nanovesicles by cells, exhibit great potential in the regeneration of bone defects and the realization of cell-free therapy. Chitosan, a natural polysaccharide, can form a thermosensitive injectable hydrogel through the addition of β-glycerophosphate. Herein, we developed injectable thermosensitive hydrogel-encapsulated sEVs derived from bone mesenchymal stem cells, which significantly prolonged delivery and release and synergistically enhanced bone regeneration. sEVs were isolated and characterized, and the physicochemical properties, release kinetics, and biocompatibility of the hydrogels were analyzed. *In vitro* experiments were performed to investigate osteogenic differentiation, cell proliferation and migration, and tube formation. Thereafter, sEVs were added to the chitosan/β-glycerophosphate hydrogel (sEV@CS/β-GP composite) to repair calvarial defects in rats. The results showed that sEV-loaded hydrogels were biocompatible, exhibiting excellent thermosensitive properties and enhancing bone regeneration. Furthermore, mechanistic studies revealed that exosomal miR-21 targeted SPRY2, thereby promoting angiogenesis. Our study provides new insights on the repair of bone defects with multifunctional controlled-sEV-release hydrogels, which shows great potential in the repair of tissues in the future.

## Introduction

Bone defects often arise from trauma, infections, tumors, congenital malformations, or skeletal diseases, and they can heal slowly or not at all, thereby leading to life-long disabilities (Zaidi, 2007; Huey et al., 2012). Currently, the “gold standard” in the treatment of bone defects is autologous and allogeneic transplantation (Crane et al., 1995; Lopes et al., 2018). In recent years, the introduction of a variety of reconstructive materials has provided new tools for orthopedic surgeons; however, the most important aspect of bone regeneration still lies in the identification of safe, effective, and aesthetic means for filling structural defects (Kawai et al., 2011; Bose et al., 2012; Zhang et al., 2019). Several studies on the modulatory processes of bone healing have demonstrated that angiogenesis plays an important role in bone regeneration. As such, bone tissue engineering combines the use of different cells, biological factors, scaffolds, and bone substitutes to improve osteogenic and angiogenic activities (Langer and Vacanti, 1993; Khademhosseini and Langer, 2016).

The use of bone mesenchymal stem cells (BMSCs) is an attractive approach to promote osteogenesis and angiogenesis in patients with bone defects (Fu et al., 2019). BMSCs can be easily harvested from donors, maintain osteogenic properties, and offer a low incidence of graft-versus-host disease. However, the direct transplantation of BMSCs associates with several challenges such as time- and dose-requirements, the low survival rate of locally transplanted cells, tumor formation, and immune rejection (Sissung and Figg, 2020). In addition, the role of BMSCs in tissue regeneration involves paracrine mechanisms, which stimulate immunomodulatory pathways, and small extracellular vesicles (sEVs) have been implicated in these processes (Li et al., 2018).

sEVs are membranous structures (diameter, 50–150 nm), namely, exosomes and microvesicles that are released from cells into the extracellular environment, and they participate in cell-to-cell communication (Tkach and Thery, 2016; van Niel et al., 2018; Xu et al., 2018). sEVs have important roles in the protection of their contents, such as mRNAs, miRNAs, and proteins, from degradation, as well as in the delivery of their contents to recipient cells, which are needed for cell function (He et al., 2018; Kalluri and LeBleu, 2020). sEVs possess stem cell-like pro-regenerative properties, and the application of sEVs may prevent many of the adverse effects of stem cell transplantation therapy. More importantly, sEVs do not contain MHC-I or MHC-II proteins; as such, they overcome the disadvantages of stem cell transplantation therapy and seldom induce overt immune reactions (Roccaro et al., 2013). Previous studies have demonstrated that BMSC-derived sEVs (BMSC-sEVs) exhibit similar or identical therapeutic roles to those of BMSCs used in the treatment of bone defects, and miRNAs may induce osteoblast differentiation and bone formation (Tan et al., 2020; Wang and Thomsen, 2021). For example, Liu et al. reported that BMSC-sEVs expressing miR-130a can stimulate the PTEN/AKT signaling pathway during angiogenesis and bone remodeling (Liu et al., 2019), whereas Liao et al. demonstrated that BMSC-sEVs expressing miRNA-122–5p can promote osteoblast proliferation during osteonecrosis of the femoral head (Liao et al., 2019b). Taken together, these findings suggest that exploring the underlying mechanisms of bone regeneration induced by exosomal miRNAs may improve our understanding of osteogenesis and angiogenesis, thereby promoting the development of new treatment strategies.

Recently, hydrogels have generated considerable interest in the field of bone repair, as they involve a minimally invasive injection and form into a solid-like object *in situ* (Ingavle et al., 2019; Kocak et al., 2020). Polymer-based hydrogels have been used in tissue repair due to their structural uniformity, biodegradability, high permeability, biocompatibility, improved mechanical strength, and application ease (Hoffman et al., 2013). Chitosan (CS), a type of endogenous polysaccharide, has been widely used due to its many properties, including antibacterial activity and exogenous biomineralization capability (Petit et al., 2020). Among chitosan-based hydrogels, chitosan (CS)/β-glycerophosphate (β-GP) hydrogels have received much interest because of their thermosensitive properties and injectability (Wasupalli and Verma, 2020). Thermosensitive hydrogels exist as liquids at room temperature but form gels at body temperature, that is, most thermally-responsive hydrogels are soluble below a specific temperature, which is known as the lower critical solution temperature (LCST), but they are insoluble above this temperature (Bhattarai et al., 2010).

To our knowledge, the effects of sEV-loaded CS/β-GP hydrogels on angiogenesis, which is critical for bone regeneration, have not yet been investigated (Wang et al., 2020). Here, an effort was made to fabricate a novel type of injectable hydrogel system (sEV@CS/β-GP composite) consisting of the CS/β-GP hydrogel and sEVs that was capable of forming a gel *in situ* at body temperature. The sEVs were released in a sustained and controlled manner, and the hydrogel was gradually degraded and internalized by human umbilical vein endothelial cells (HUVECs) and human BMSCs. Furthermore, the absorbed sEVs exhibited osteogenic and angiogenic properties. Previous studies have also reported that angiogenic activity was also promoted by miR-21 overexpression in BMSC-sEVs (Wu et al., 2020; Li G.-Q. et al., 2021; Zhang et al., 2021). In summary, sEV@CS/β-GP hydrogels can promote bone regeneration, which was likely mediated by miR-21 overexpression. These findings provide new insights on a promising therapeutic strategy for cell-free bone repair.

## Materials and Methods

### Cell Culture

BMSCs, HUVECs, and HEK-293 cells were obtained from the cell bank of the Chinese Academy of Medical Sciences (Beijing, China). BMSCs were cultured in basal media (Cyagen Biosciences, Santa Clara, CA, United States), and HUVECs and HEK-293 cells were cultured in high-glucose Dulbecco’s modified Eagle’s medium (DMEM, Gibco BRL, Grand Island, NY, United States) supplemented with 10% fetal bovine serum (FBS) and 1% penicillin–streptomycin.

### Isolation and Characterization of sEVs

#### sEV Isolation and Purification

sEVs were isolated and purified from BMSC supernatants by ultracentrifugation as previously described (Colombo et al., 2014). Before isolation, BMSCs were incubated for 48 h in medium supplemented with 10% sEV-depleted FBS (Umibio, Shanghai, China). The supernatant was collected and centrifuged at 300 × g for 10 min, 2000 × g for 20 min, and 10,000 × g for 30 min to discard the cell debris, and centrifuged at 100,000 × g for 70 min to collect the sEVs. The pelleted sEVs were washed two times with PBS and centrifuged at 110,000 × g for 70 min to remove the contaminating proteins. All procedures were performed at 4°C, and the sEVs were resuspended in PBS.

#### sEV Identification and Internalization

The size distribution of sEVs was determined by nanoparticle tracking analysis (NTA) with the NanoSight NS500 system (Malvern Instruments, Malvern, United Kingdom), and the morphology of sEVs was observed by transmission electron microscopy (TEM, Hitachi, Tokyo, Japan) as previously described (Wu et al., 2021). Western blotting was used to detect the expression of sEV-specific surface markers, including CD63, CD81, and TSG101.

The uptake of sEVs by BMSCs and HUVECs was examined by labeling sEVs with the fluorescent dye PKH26 or PKH67 (Sigma-Aldrich, Darmstadt, Germany), according to the manufacturer’s instructions, which were then incubated with BMSCs or HUVECs at 37°C for 24 h. Subsequently, the cells were fixed with 4% paraformaldehyde, stained with DAPI for 10 min, and observed by confocal microscopy (Nikon, Tokyo, Japan).

### Impact of sEVs on Osteogenic Differentiation

#### Alizarin Red Staining (ARS) and Alkaline Phosphatase (ALP) Activity

The osteogenic differentiation of BMSCs was carried out 24 h after the incubation of sEVs or transfection of miRNA mimic or inhibitor. Briefly, the medium was replaced with osteogenic differentiation medium (Cyagen Biosciences), which was refreshed every 72 h, supplemented with PBS (200 μL) or different concentrations of BMSC-sEVs (50 or 100 μg/ml). To assess mineralization, ARS was performed on day 14 after osteoinduction. Cells were stained with 2% ARS solution (Sigma-Aldrich) for 10 min and then washed with distilled water. To quantitatively determine matrix calcification, the cells were de-stained with 10% cetylpyridinium chloride in 10 mM sodium phosphate for 30 min, and the absorbance was measured at 562 nm.

To assess ALP activity, the ALP assay kit (Beyotime, Jiangsu, China) was used. BMSCs were cultured in osteogenic differentiation medium and lysed on days 7 and 14 with 0.1% Triton X-100 in Tris-HCl for 2 h at 4°C. *p*-Nitrophenyl phosphate was added to the cell lysates, and the samples were incubated at 37°C for 15 min. The normalized ALP activity was obtained by determining the total intracellular protein concentration with the Pierce BCA Protein Assay kit (Thermo Fisher Scientific, Waltham, MA, United States).

### Impact of sEVs on Angiogenesis

#### Cell Proliferation Assay

Three groups were prepared according to the BMSC-sEVs concentration (0, 50, and 100 μg/ml), with each group consisting of quadruplicate wells. HUVECs (5 × 10^3^ cells/well) were inoculated into 96-well plates and treated with the different BMSC-sEVs concentrations. The medium was changed every 2 days. To assess cell viability, the Cell Counting kit-8 (CCK-8; Dojindo, Tokyo, Japan) was used. On days 1, 3, 5, and 7, the absorbance was measured at 450 nm, and the growth curve was generated. CCK8 assay was also performed to test the promoting effect of gradient concentrations (400, 200, 100, 50, 25, and 0 μg/ml) of sEVs on cell proliferation.

#### Cell Migration Assay

HUVECs (1 × 10^4^ cells/well) were resuspended in serum-free medium and seeded into the upper chamber of Corning 8-μm pore size transwell units (Corning, NY, United States) that were housed in 24-well plates. The lower chamber was filled with DMEM supplemented with 10% sEV-depleted FBS that was pre-incubated with PBS (200 μL), BMSC-sEVs (50 μg/ml), or BMSC-sEVs (100 μg/ml). The plates were incubated at 37°C for 24 h. Thereafter, the cells attached to the upper surface of the filter membranes were removed, and those attached to the lower surface were stained with 0.1% crystal violet. Cell migration was observed by light microscopy (Leica, Solms, Germany).

#### Tube Formation Assay

The *in vitro* angiogenesis assay was conducted using Matrigel basement membrane matrix (BD Biosciences, San Jose, CA, United States), according to the manufacturer’s instructions. Briefly, Matrigel was thawed overnight at 4°C and added to 96-well plates (50 μL/well), which were then incubated at 37°C. Thereafter, HUVECs (2 × 10^4^ cells/well) were resuspended in complete medium supplemented with 10% sEV-depleted FBS pre-incubated with PBS (10 μL), BMSC-sEVs (50 μg/ml), or BMSC-sEVs (100 μg/ml). The plates were incubated at 37°C for 6 h, and tube formation was observed by inverted microscopy. Five fields from each well were randomly selected, and the total tube length was measured with ImageJ software (National Institutes of Health, Bethesda, MD, United States).

#### Quantitative Real-Time PCR (RT-qPCR) Analysis

TRIzol (Invitrogen, Carlsbad, CA, United States) was used to isolate total RNA, and the Revert Aid First-Strand cDNA Synthesis kit (Vazyme, Jiangsu, China) was used to reverse transcribe RNA, according to the manufacturer’s instructions. The sEV RNA Purification kit (Umibio, Shanghai, China) was used to extract miRNA, and the SYBR Green microRNA Assay kit (Applied Biosystems, Foster City, CA, United States) was used to synthesize cDNA. Real-time PCR was performed with the ABI PRISM 7900 H T system using the SYBR Green Master-Mix kit (Applied Biosystems). GAPDH and U6 were used to normalize mRNA and miRNA expression levels, respectively, and the 2^−ΔΔCt^ method was used to quantify the relative expression levels. All primer sequences are listed in Table 1.

**TABLE 1:** List of primers used.

Gene	Primer sequence, 5′–3′	
	Forward	Reverse
*OCN*	CAC​TCC​TCG​CCC​TAT​TGG​C	CCC​TCC​TGC​TTG​GAC​ACA​AAG
*OPN*	GAA​GTT​TCG​CAG​ACC​TGA​CAT	GTA​TGC​ACC​ATT​CAA​CTC​CTC​G
*Runx2*	TCA​ACG​ATC​TGA​GAT​TTG​TGG​G	GGG​GAG​GAT​TTG​TGA​AGA​CGG
*VEGF*	AGC​GCC​GAA​GTC​CAG​AAA​AC	AGG​GTC​TCG​ATT​GGA​TGG​CA
*bFGF*	AGA​AGA​GCG​ACC​CTC​ACA​TCA	CGG​TTA​GCA​CAC​ACT​CCT​TTG
*ANG-1*	AGC​GCC​GAA​GTC​CAG​AAA​AC	TAC​TCT​CAC​GAC​AGT​TGC​CAT
*GAPDH*	CAG​GGC​TGC​TTT​TAA​CTC​TGG	TGG​GTG​GAA​TCA​TAT​TGG​AAC​A
*U6*	TGG​AAC​GCT​TCA​CGA​ATT​TGC​G	GGA​ACG​ATA​CAG​AGA​AGA​TTA​GC

#### Western Blotting

The total protein concentrations in cells and sEVs were determined using the BCA Protein Assay kit. Proteins were separated by 10% SDS-PAGE, transferred to PVDF membranes, and probed with the appropriate primary and secondary antibodies. Immunoreactive bands were visualized using enhanced chemiluminescence reagents (Thermo Fisher Scientific) and quantified using ImageJ software. Primary antibodies against CD63, CD81, TSG101, Calnexin, OCN, OPN, Runx2, VEGF, bFGF, ANG-1, and CD31 were obtained from Abcam (Cambridge, United Kingdom).

### Preparation and Characterization of Hydrogels

#### Hydrogel Preparation

Thermosensitive CS/β-GP hydrogels were prepared as previously described (Wu et al., 2019). Briefly, 0.2 g of CS (degree of deacetylation, 95%; Sigma-Aldrich) was added to an acetic acid solution (0.1 mol/L, 10 ml) to obtain the 2% (w/v) CS solution, followed by the dropwise addition of 1 ml of 56% (w/v) β-GP solution (Merck, Darmstadt, Germany) while stirring.

#### Thermosensitivity

The sol-to-gel transition behavior of the hydrogels at 37 °C was determined by the test tube inversion method (Park et al., 2012), which uses flow (sol) or no-flow (gel) criteria to assess flow when sample tubes are inverted for 30 s at a controlled temperature. The gelation time was determined by inverting the vial every 30 s. Five independent duplicate tests were performed. The gelation time was recorded, and the average value was used.

#### Scanning Electron Microscopy (SEM)

Hydrogels were frozen at −80°C for 24 h and lyophilized at −40°C for 48 h in a freeze-dryer. Thereafter, the samples were cut with a sharp blade to obtain cross-sections and sputter-coated with a gold-platinum layer. Cross-sections were observed by SEM (XL30, PHILIPS, Eindhoven, Netherlands). The pore size was analyzed by ImageJ software, and the average pore size was calculated based on 50 pores in five randomly selected areas per sample.

#### Viscosity

A rheometer (Kinexus Ultra, Malvern, United Kingdom) equipped with a parallel plate with a diameter of 20 mm and a gap distance of 0.5 mm was used to measure the rheological properties of the hydrogel solution. The variations in the storage (G′) and loss (G″) moduli of the samples were measured under constant strain (0.1%) and frequency (1.0 Hz). The hydrogel solution was added to the parallel plate using a temperature range of 25–45°C and a heating rate of 0.5°C/min.

#### Swelling and Degradation Behaviors

Hydrogels (n = 3) were dried, weighed (W_d_), and rehydrated in PBS for 24 h at 37°C. Samples were removed from PBS and weighed again (W_s_) at different time points after blotting the surface with filter paper (Yuan et al., 2019). The swelling ratio was calculated as follows (W_s_ – W_d_)/W_d_ × 100%

The degradation of the hydrogels was reflected in the weight loss, which was also studied in PBS at 37°C. At given time points (days 1, 4, 7, 10, 14, and 21) after blotting the surface with filter paper, the hydrogel weight was measured. The weight loss ratio was defined as follows (W_0_ – W_t_)/W_0_ × 100%, where W_0_ is the initial weight of the sample, and W_t_ is the weight of the sample at a specific time point.

#### Mechanical Properties Test

The mechanical properties of the hydrogels were determined with a universal mechanical analyzer (Instron, Norwood, MA, United States). Photo-crosslinked cylindrical hydrogels (n = 4) with a height of 5 mm and a diameter of 10 mm were placed on the lower plate at a speed of 1 mm/min, and the compressive force was recorded until the hydrogels were deformed by the upper plate.

#### Ability of sEVs/Gel Composites to Release sEVs

sEVs (1 × 10^8^ particles/mL) were added to the composited hydrogels, and the release of sEVs from hydrogels was measured using the BCA Protein Assay kit. Briefly, the hydrogels were immersed in PBS in 24-well plates. At specific time points, the liquid on the surface of the hydrogels was collected, and the PBS in the wells was replaced. sEVs release was quantified and expressed as a percentage. Data were presented as mean ± SD of three replicates.

### *In Vivo* Animal Experiments

#### Surgical Procedures

All procedures were approved by the Animal Research Committee of Peking Union Medical College Hospital (XHDW-2020-040), and all surgical procedures were performed in a sterile environment. Rats were anesthetized with an intraperitoneal injection of pentobarbital sodium (50 mg/kg). Thereafter, a 1.0–1.5-cm midline sagittal incision was made on the scalp, and the calvarium was exposed by blunt dissection. Two full-thickness critical-size calvarial defects with a diameter of 5 mm each were created with a drill with a sterile bit, and 18 male rats at 8 weeks-of-age were randomly allocated into three groups as follows: PBS (control), CS/β-GP hydrogel, and CS/β-GP hydrogel with 200 μg sEVs (sEV@CS/β-GP). Thereafter, the bone defects were closed and sutured with degradable silk thread. All rats were housed individually and provided food and water in a temperature-controlled environment with regular use of prophylactic antibiotics.

#### Micro-CT Analysis

Rats were euthanized at 12 weeks after surgery, and the skulls were explanted and fixed in 4% (w/v) paraformaldehyde. The morphology of the skulls was assessed using micro-computed tomography (CT) to determine the bone volume. The percentage of new bone volume to tissue volume (BV/TV), bone mineral density (BMD), trabecular number (Tb.N), and trabecular thickness (Tb.Th) were determined using Mimics software (Materialise, Leuven, Belgium).

#### Histological, Immunohistochemical, and Immunofluorescence Analysis

Specimens were fixed with 4% (w/v) paraformaldehyde solution, decalcified with 5% (w/v) EDTA, dehydrated in a graded series of alcohol solutions, and embedded in paraffin. Thereafter, 5-μm-thick cross-sections from mid-defect cranial regions were stained with hematoxylin and eosin (H&E) and observed by light microscopy. Masson’s trichrome staining was used to evaluate the degree of collagen maturation.

For immunohistochemical analysis, the cross-sections were treated with antigen retrieval buffer and incubated with a CD31 primary antibody (1:100) at 4°C overnight. Thereafter, the cross-sections were incubated with a secondary antibody (1:250) at room temperature, followed by staining with DAB and counterstaining with hematoxylin. CD31 immunofluorescence staining was performed to examine new capillary formation. Cross-sections were incubated a CD31 primary antibody (1:100) at 4 °C overnight and a secondary antibody (1:250) at room temperature for 1 h in the dark.

### Exosomal miR-21-Mediated Angiogenesis by Targeting SPRY2

#### Luciferase Reporter Assay

The 3′untranslated region (UTR) of wild-type (wt) and mutant (mut) SPRY2 was amplified by PCR and individually inserted into the pGL3 plasmid. HEK293 cells (5 × 10^4^) were seeded in 48-well plates and co-transfected with the wt or mut luciferase reporter (100 ng) and miR-21 mimics (20 nM) or negative control (NCs) as indicated. At 48 h after transfection, the relative luciferase activity was detected with the Bright-Glo luciferase Assay system (Promega, Madison, WI, United States).

#### Cell Transfection

To examine the function of miR-21, HUVECs were transfected with miR-21 mimic or inhibitor and the respective NC (RiboBio, Guangzhou, China) using Lipofectamine 3000 reagent (Invitrogen). For SPRY2 overexpression, HUVECs were transfected with SPRY2 cDNA (Genechem, Shanghai, China) using Lipofectamine 3000 reagent.

#### Statistical Analysis

All experiments were performed at least in triplicate. All the data are presented as the mean ± standard deviation (SD). Multiple group comparisons were performed by two-way analysis of variance with Tukey’s post hoc test. Statistical analysis was carried out with GraphPad Prism 7.0 (GraphPad Software, La Jolla, CA, United States), and statistical significance was taken at (*) *p* < 0.05 and (**) *p* < 0.01.

## Results

### Characterization of sEVs

**FIGURE 1:**
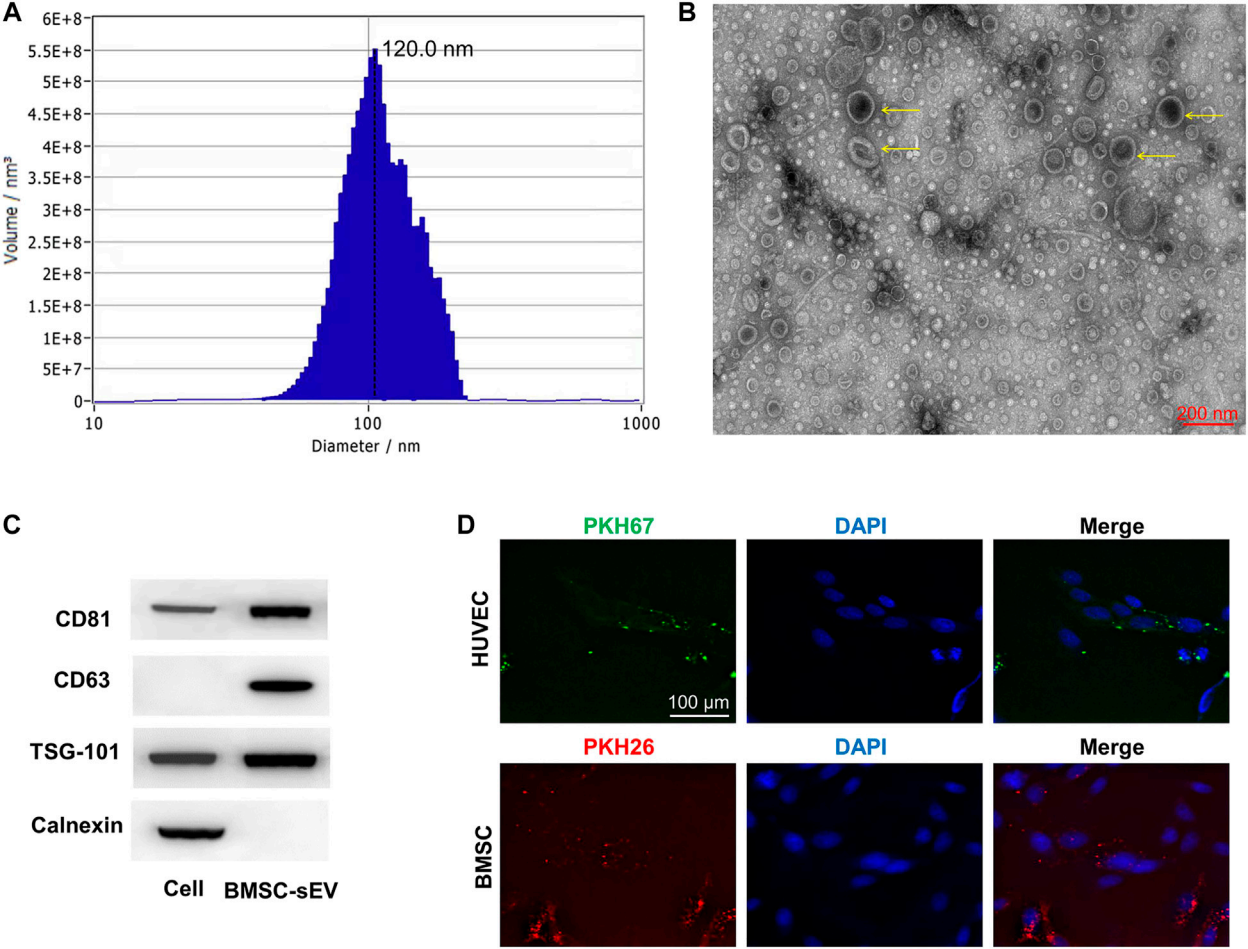
Characterization and internalization of sEVs **(A)** Particle size distribution of sEVs detected by NTA and the mean diameter is 120.0 nm **(B)** Representative images of the morphology of sEVs by TEM. The yellow arrows indicate sEVs. Scale bar = 200 nm **(C)** Western blotting analysis of the exosomal proteins CD63, CD81, TSG101 and the negative marker Calnexin **(D)** Uptake of green fuorescent dye (PKH67)-labeled and red fuorescent dye (PKH26)-labeled sEVs by HUVECs and BMSCs, respectively.

sEVs were successfully isolated from BMSCs, and BMSC-sEVs were characterized by NTA analysis, TEM analysis, and western blotting. NTA analysis revealed the size distribution of the sEVs to consist of a single bell-shaped curve with a peak at approximately 120.0 nm, and the percentage of the sEVs with a diameter in the range of 30–150 nm was >99% (Figure 1A). TEM images showed the sEVs to be round or cup-shaped with a diameter in the range of 100–150 nm (Figure 1B). Western blotting analysis (Figure 1C) showed that sEVs, and not BMSCs, expressed CD63, CD81, and TSG101 but not calnexin (negative control). Representative fluorescence microscopy images (Figure 1D) showed that sEVs were observed in the cytoplasm, which was indicative of their attachment and internalization by BMSCs and HUVECs. In addition, immunoreactive PKH26 (red) and PKH67 (green) were observed around the nucleus (blue).

### Pro-Osteogenic and Pro-angiogenic Effects of BMSC-sEVs *in vitro*

**FIGURE 2:**
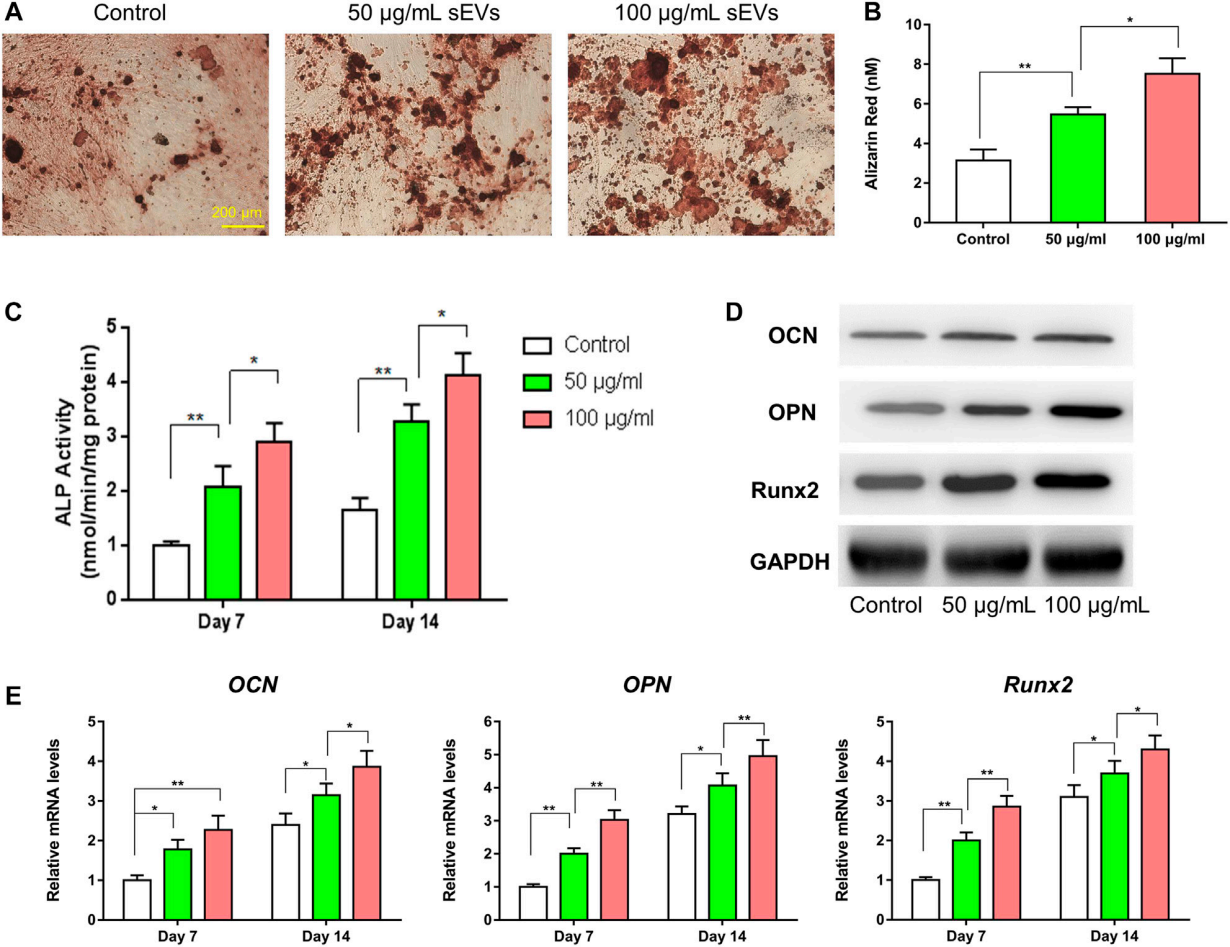
Enhanced osteogenic effect of sEVs *in vitro* (*) *p* < 0.05 (**) *p* < 0.01 **(A)** ARS results from stimulation of sEVs on BMSCs after 14 days of culture. Scale bar = 200 μm **(B)** Quantitative analysis of ARS **(C)** Quantifcation of ALP staining after incubation of BMSCs with sEVs for 7 and 14 days **(D)** Western blotting assay for the protein expression of OCN, OPN, and Runx2 **(E)** mRNA level of pro-osteogenic-related genes (*OCN*, *OPN*, and *Runx2*) in BMSCs exposed to different treatments.

The promoting proliferative effect was enhanced as the concentrations of sEVs increased, while this effect was significantly reduced or faded when sEVs were more than 200 μg/ml (Supplementary Figure S1). Compared with controls, the results of ARS showed that the different concentrations of sEVs could enhance mineral deposition by BMSCs on day 14, and the highest concentration of sEVs could induce matrix mineralization and calcified nodule formation (Figure 2A). In quantitative analysis, there was significantly more calcium accumulation in the group treated with sEVs at 100 μg/ml than that in the group treated with sEVs at 50 μg/ml or the untreated group (*p* < 0.05; Figure 2B). ALP activity, an indicator of early-stage osteogenic differentiation of BMSCs, was significantly higher in cells exposed to both concentrations than that in control cells on days 7 and 14 (*p* < 0.05; Figure 2C), and the effects were dose-dependent. The mRNA levels of *OCN*, *OPN*, and *Runx2* gradually increased in cells exposed to sEVs from days 7–14, and the protein levels of OCN, OPN, and RUNX2 significantly increased on day 14 (Figure 2D), indicating that BMSC-sEVs can upregulate the expression of osteogenic genes (*p* < 0.05). The most significant increases in mRNA expression were observed in cells treated with the highest concentration of sEVs (Figure 2E), revealing that the effects were dose-dependent.

**FIGURE 3:**
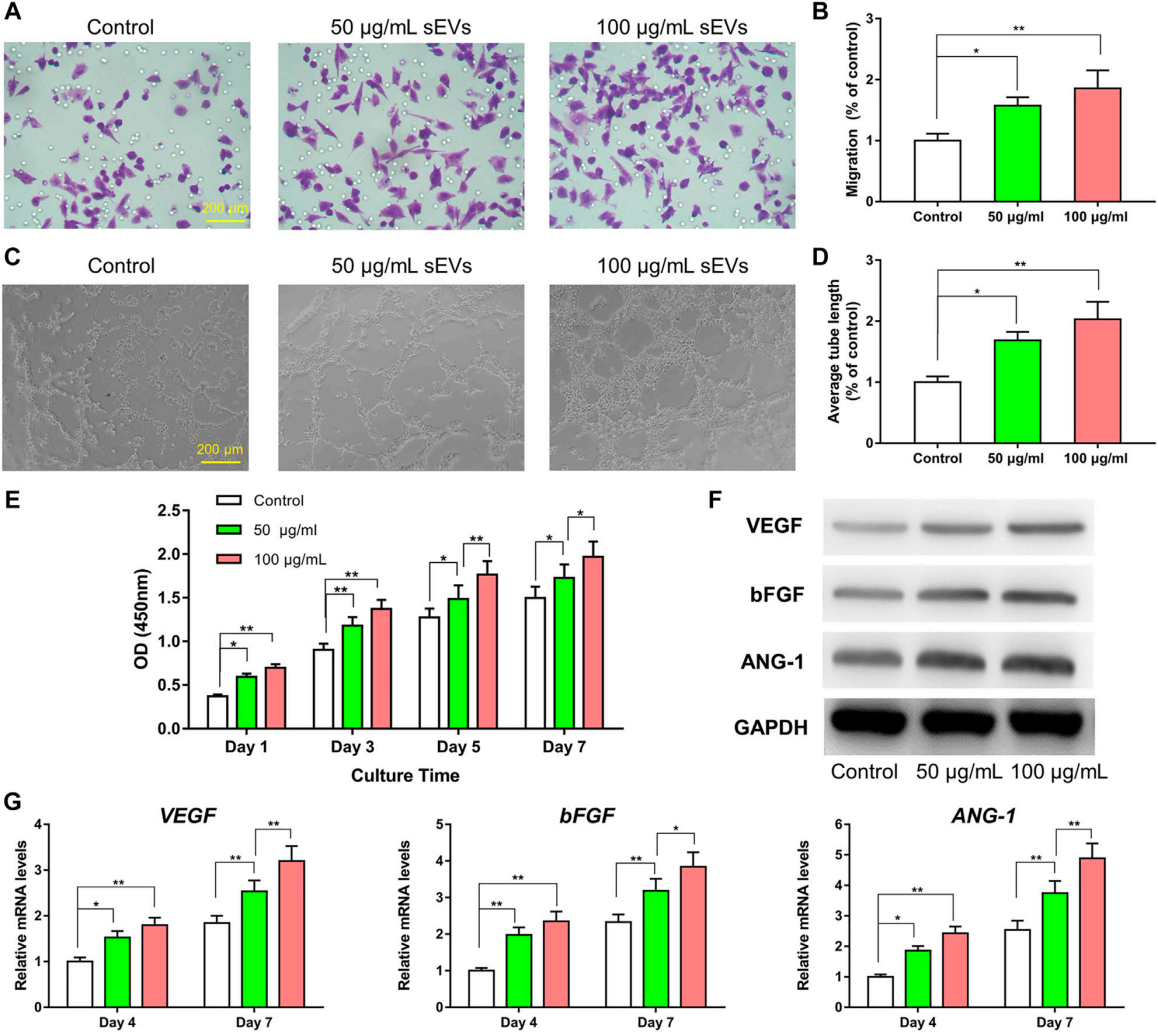
Enhanced angiogenic effect of sEVs *in vitro* (*) *p* < 0.05 (**) *p* < 0.01 **(A)** Transwell assay and quantitative analysis **(B)** of the cell migration rate **(C)** Tube formation by HUVECs and quantitative analysis **(D)** of the average tube length **(E)** CCK8 assay for the proliferation of HUVECs exposed to different treatments **(F)** Western blotting assay for the protein expression of VEGF, bFGF, and ANG-1 **(G)** mRNA level of pro-angiogenic-related genes (*VEGF*, *bFGF*, and *ANG-1*).

As shown in Figures 3A–D, the migratory capability of HUVECs exposed to sEVs for 24 h was greater than that in control cells, and the effects were dose-dependent. Furthermore, tube formation was increased in cells treated with sEVs at a concentration of 100 μg/ml compared with those treated at a concentration of 50 μg/ml or PBS. Compared with the control group, the proliferation of HUVECs exposed to BMSC-sEVs at both concentrations was also increased on days 1, 3, 5, and 7 (*p* < 0.05), and the effects were dose-dependent (Figure 3E). Compared with controls, the protein levels of VEGF, bFGF, and ANG-1 increased in cells exposed to sEVs, with the highest concentration showing the most significant changes (Figure 3F). Likewise, compared with controls, the mRNA levels of *VEGF*, *bFGF*, and *ANG-1* increased in cells exposed to sEVs on days 4–7, and the effects were dose-dependent (Figure 3G). These findings indicate that BMSC-sEVs can dose-dependently promote angiogenesis.

### Characterization of sEV Release From CS/β-GP Hydrogels

**FIGURE 4:**
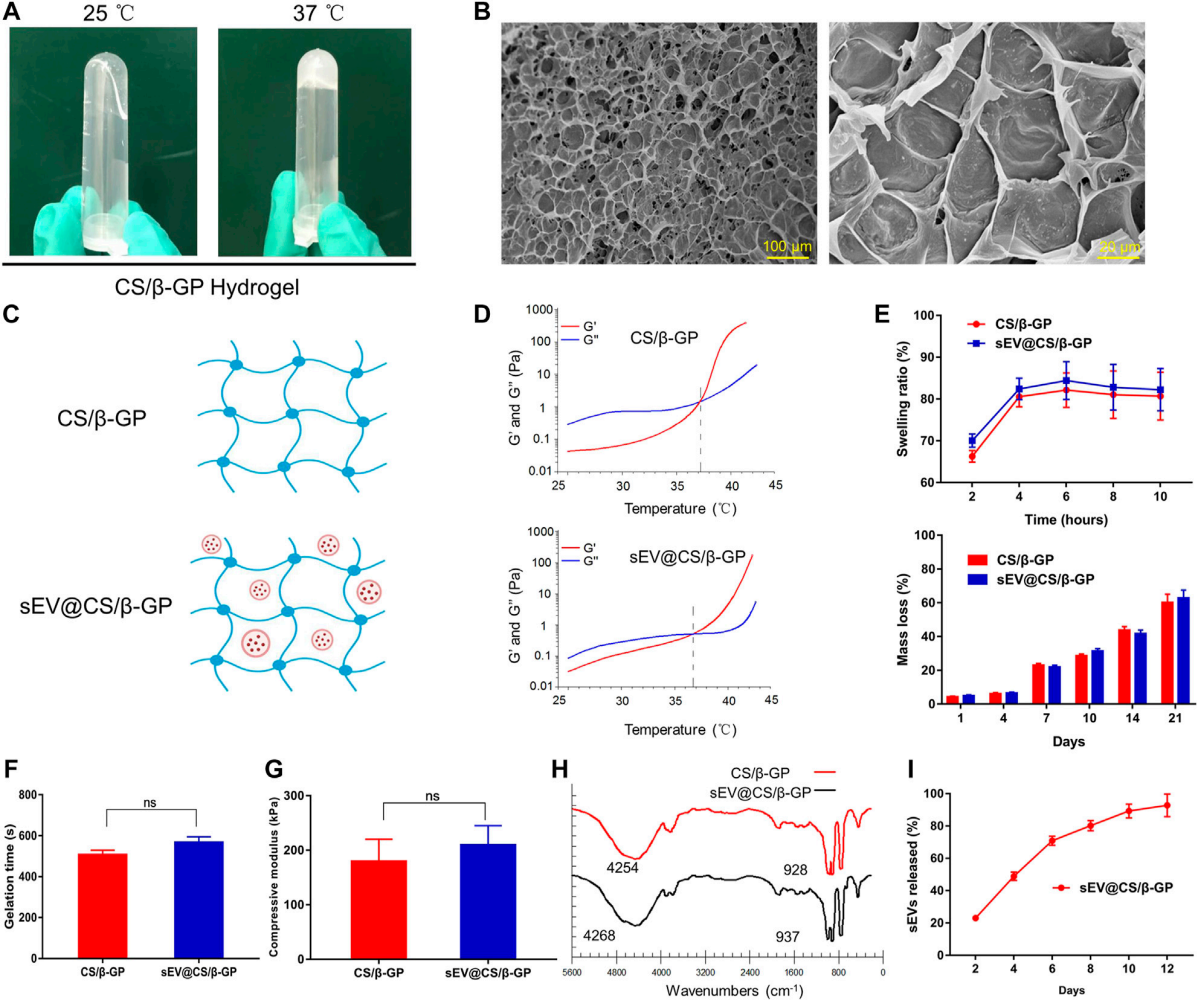
Characterization and the sEV release ability of the CS/β-GP hydrogel. ns: no significant difference **(A)** Sol-to-gel transition: a photograph of CS/β-GP thermosensitive hydrogel at 25 and 37°C **(B)** SEM photomicrographs of the morphological structure of CS/β-GP hydrogel **(C)** Schematic representation of the combination of sEVs and CS/β-GP hydrogel **(D)** Typical temperature-dependent functions of G′ and G″ for CS/β-GP and sEV@CS/β-GP hydrogels **(E)** Swelling rate and mass-remaining profiles of CS/β-GP and sEV@CS/β-GP hydrogels **(F)** Gelation time of CS/β-GP and sEV@CS/β-GP hydrogels **(G)** The compressive module of CS/β-GP and sEV@CS/β-GP hydrogels **(H)** FTIR (Fourier-transform infrared) spectra of CS/β-GP and sEV@CS/β-GP hydrogels **(I)** release profiles of sEVs from the sEV@CS/β-GP hydrogel.

As shown in Figure 4A, the CS/β-GP hydrogel was a colorless and transparent liquid at room temperature (25°C). It underwent a sol-to-gel transition as the temperature increased, and at physiological temperature (37°C), the CS/β-GP hydrogel transformed into a non-flowing hydrogel. SEM results revealed that the hydrogel was porous and encased in a dense, thick polymeric wall (Figure 4B). Figure 4C shows the process by which hydrogels were loaded with sEVs. To verify the sol-to-gel transition, the viscosity was examined as a function of the temperature. The hydrogels had low elastic (G′) and viscous (G″) moduli at 25°C and the intersection of G′ and G″, which was characterized as G′ < G″ (Figure 4D), indicating their viscous nature, while the intersection of G′ and G″ (G′ = G″) clearly revealed their gelation. Furthermore, the gap between the G′ curve and the G″ curve for sEV-loaded hydrogels was slightly smaller compared with unloaded hydrogels. These results indicate that the hydrogel behaved as a liquid before gelation and as a gel after gelation, that is, at physiological temperature and pH.

Both CS/β-GP and sEV@CS/β-GP had good biodegradability and swelling behavior (Figure 4E). The swelling ratio of the hydrogel increased with time, reaching equilibrium at approximately 6 h, and there was no significant difference between the two hydrogels. Likewise, there was no difference in the weight loss of the two hydrogels, and the hydrolysis of imine bonds within the hydrogel in PBS was the likely mechanism. As shown in Figure 4F, there was a slight but non-significant increase in the gelation time when sEVs were added to CS/β-GP hydrogels (508 ± 21 and 569 ± 26 s). The maximal stress and strain of hydrogels were obtained from compressive curves, and the results indicated that both hydrogels had similar maximal stress and strain (Figure 4G). FTIR spectra revealed the addition of sEVs did not cause significant structural changes (Figure 4H), as the sEVs may have interacted with certain functional groups to reduce the formation of hydrogen chemical bonds. A slight increase in wavelengths (from 4,254 to 4,268 cm^−1^ and 928 to 937 cm^−1^) indicated that sEVs had no impact on the thermosensitive hydrogels. Moreover, the sEV@CS/β-GP hydrogel showed good slow-release performance (Figure 4I), with 80% of the sEVs releasing on day 8 and the release rate slowing after this time point.

### BMSC-sEVs Promote the Repair of Calvarial Defects *in vivo*

**FIGURE 5:**
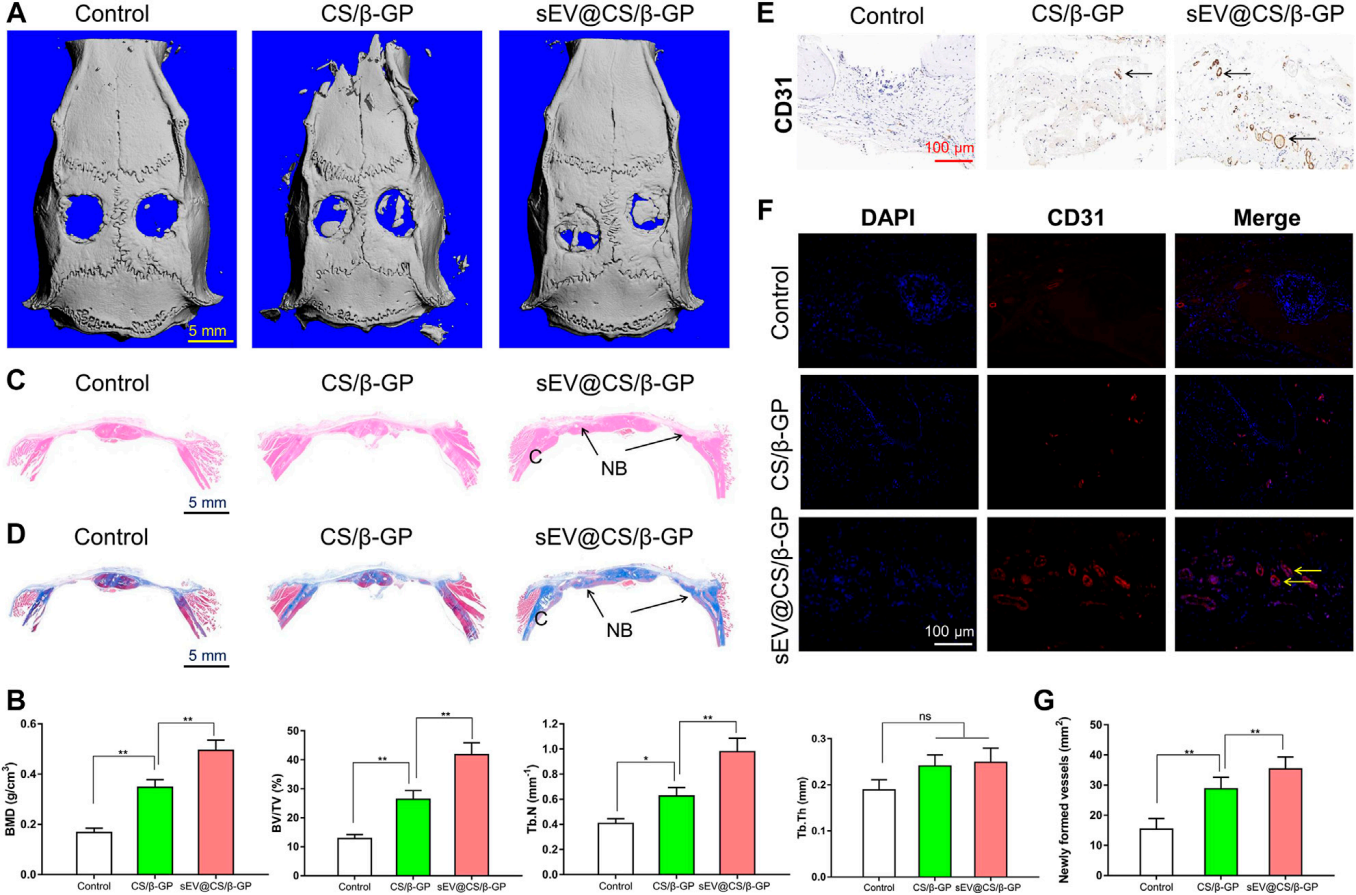
sEV@CS/β-GP hydrogel promoted bone formation in critical-sized rat calvarial defects (*) *p* < 0.05 (**) *p* < 0.01, ns: no significant difference **(A)** Micro-CT images of bone defects in each group after 12 weeks. Scale bar = 5 mm **(B)** Quantitative analysis of BMD, BV/TV ratio, Tb.N and Tb.Th in the diferent groups **(C)** H&E staining and Masson’s trichrome staining **(D)** in the three groups. C, cranium. NB, new bone **(E)** Immunohistochemical staining and immunofuorescence analysis **(F)** of the angiogenic marker CD31. Dark brown granules indicating positive staining are marked by black arrows, and yellow arrows mark the newly formed vessels **(G)** Quantitative analysis of newly formed vessels.

The 3D reconstruction of micro-CT images of rat calvarial defects at 12 weeks are shown in Figure 5A. Compared with the control group, the formation of new bone, which filled the calvarial defects, was observed in CS/β-GP and sEV@CS/β-GP groups, with the sEV@CS/β-GP group showing a greater area of newly formed bone. The repair of these bone defects was further examined using quantitative approaches, and the BMD, BV/TV ratio, and Tb.N in CS/β-GP and sEV@CS/β-GP groups were all significantly higher than those in the control group, indicating that the release of sEVs from hydrogels and the thermosensitive property of hydrogels improved the bone healing capacity, whereas there was no difference in the Tb.Th (Figure 5B). The results of histological staining indicated that the bone defects in the control group were mainly filled with fibrotic connective tissue, whereas newly formed bone was observed both along the border and in the center of the calvarial defects after application of CS/β-GP hydrogels, with sEV@CS/β-GP hydrogels showing a greater area of newly formed bone (Figures 5C,D, Supplementary Figure S2,3). These results were similar to those of micro-CT analysis. The results of CD31 immunohistochemical staining of right bone defect sections indicated that there were more CD31-positive cells in the sEV@CS/β-GP group compared with the CS/β-GP group, which was indicative of new vessel formation within the bone defect (Figure 5E), which was further verified by quantitative analysis (Figures 5F,G). These results reveal that sEVs promoted calvarial defect repair and enhanced angiogenesis and osteogenesis.

### BMSC-Derived Exosomal miR-21 Promotes Angiogenesis by Targeting SPRY2

**FIGURE 6:**
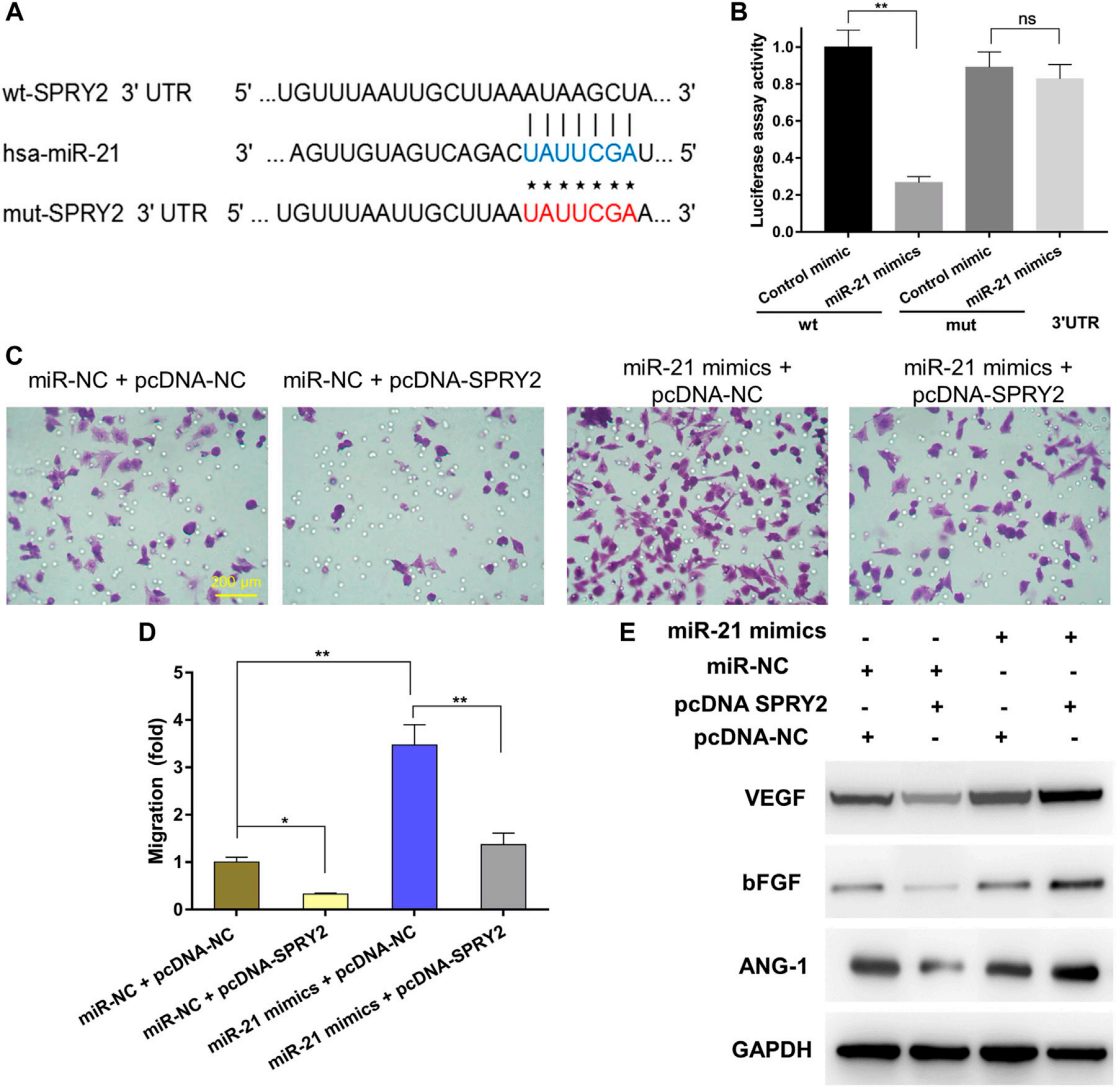
Exosomal miR-21 promoted angiogenesis by targeting SPRY2 (*) *p* < 0.05 (**) *p* < 0.01, ns: no significant difference. wt, wild-type; mut, mutant; NC, negative control **(A)** The miR-21 binding sequence in the 3ʹ-UTR of SPRY2 **(B)** The miR-21 mimics transfection reduced luciferase activity when compared to control mimics transfection, which confirms that SPRY2 are the target genes of miR-21 **(C)** The transwell assay showing the cell migration when HUVECs were co-transfected with miR-21 mimics and pcDNA-SPRY2, and the quantitative analysis **(D)** indicated overexpression of SPRY2 suppressed the upregulation of the migration rate of HUVECs by miR-21 mimics **(E)** Western blotting assays showing that overexpression of SPRY2 prevented the upregulation of VEGF, bFGF, and ANG-1 protein expression by miR-21 mimics.

Previous studies have reported that miR-21 is expressed at a high level in sEVs derived from BMSCs (Lv et al., 2017). As such, we analyzed miRNA expression in BMSCs using an existing GEO dataset (GSE78865) and predicted the candidate target genes of miR-21 that contributed to angiogenesis by exploring online databases, including TargetScan, miRanda and miRWalk. KEGG pathway enrichment analysis was performed for candidate target genes related to angiogenesis. To confirm the direct binding between miR-21 and the 3ʹ-UTR of its predicted target gene SPRY2, we performed reporter assays using a luciferase reporter plasmid containing the wt or mut SPRY2 3ʹ-UTR with the miR-21 binding site (Figure 6A). Transfection of HUVECs with the miR-21 mimics could reduce luciferase activity compared to transfection with the control mimic, which was indicative of direct binding between miR-21 and the SPRY2 3-ʹUTR (Figure 6B).

To further explore the relationship between exosomal miR-21 and SPRY2, rescue experiments were conducted. We transfected the miR-21 mimics or miR-NC into HUVECs, followed by co-transfection with a SPRY2-overexpressing plasmid (pcDNA-SPRY2). The results revealed that the migratory capacity of cells co-transfected with the miR-21 mimics and pcDNA-SPRY2 was enhanced compared with cells co-transfected with the miR-NC and pcDNA-SPRY2 (Figures 6C,D). The levels of angiogenic proteins in cells transfected with the miR-21 mimics were higher than those in control cells, and pcDNA-SPRY2 could abolish the effect of the miR-21 mimics on angiogenesis (Figure 6E), indicating that exosomal miR-21 can promote HUVEC migration and angiogenesis by targeting SPRY2.

## Discussion

Angiogenesis and osteogenesis are highly coupled processes that are indispensable for bone repair (Kusumbe et al., 2014). Large bone defects caused by trauma and certain diseases may not heal naturally and require regenerative scaffold implantation to promote tissue reconstruction (Dimitriou et al., 2011). Although a variety of bioengineering techniques that promote tissue regeneration with optimized materials are currently available, vascularization after scaffold implantation is still a major challenge (Potente et al., 2011; Rouwkema and Khademhosseini, 2016; Eelen et al., 2020). The osteogenic function of cells that promote bone regeneration requires a network of microvessels, which mediate the transport of circulating of cells, oxygen, nutrients, and waste products (Laschke and Menger, 2015). Besides, Zhao et al. modified the tetrahedral framework nucleic acid (tFNA) with aptamers to form aptamer-tFNA nanostructures, tFNA-Apt02 and tFNA-AptVEGF, and they exhibited stronger angiogenesis, further provided a new and efficient proangiogenic approach (Zhao et al., 2021). Therefore, newly formed microvessels within grafts are critical for successful bone tissue engineering.

Mesenchymal stromal/stem cells (MSCs) have been widely applied because they can be obtained easily from adult tissues, as well as proliferate and differentiate into bone, adipose, or cartilage (Grayson et al., 2015). However, the direct use of MSCs for therapeutic purposes remains limited by many risk factors, such as tumor formation, thrombosis, and unwanted immune responses. Furthermore, the stimulation of localized healing by MSCs involves paracrine mechanisms (Gnecchi et al., 2005; Caplan and Correa, 2011), and the application of sEVs may overcome these limitations. Because of their ideal characteristics, sEVs are also favorable nanoscale drug carriers for the regeneration of tissues and the treatment of certain diseases (Liao et al., 2019a; Elsharkasy et al., 2020). For example, Dong et al. revealed that the use of fetal bovine serum-derived sEVs to carry Icariin could promote osteoblast proliferation and bone regeneration more effectively than Icariin alone (Dong et al., 2021). Wu et al. demonstrated that BMSC-derived sEVs and Fe_3_O_4_ nanoparticles under conditions of a static magnetic field could facilitate bone regeneration and enhance wound healing (Wu et al., 2020; Wu et al., 2021). In the present study, we manufactured thermosensitive hydrogels loaded with BMSC-derived sEVs that could promote bone regeneration.

Hydrogels have been widely used in tissue bioengineering, drug delivery, and cell-based therapy. A recent study reported a biphasic hydrogel for osteochondral defect regeneration, which was fabricated via a thermally reactive, rapid cross-linking method (Liao et al., 2017). CS is an excellent excipient because it is non-toxic, stable, biodegradable, and sterilizable, which makes it a versatile material with application potential in biomedical and biotechnological fields (Kumar et al., 2004). CS can form a thermosensitive injectable hydrogel through cross-linking with β-GP via ionic interactions between the ammonium groups of CS and the phosphate groups of β-GP, which can increase the gelation temperature and pH to the physiological range and restrict the immediate precipitation/deformation of the hydrogel (Bhattarai et al., 2010). The thermosetting of hydrogels has an added advantage, that is, once the homogeneous solution is injected into the tissue defect, the hydrogel forms and strengthens mechanically with time. In addition, the ability of polymer matrix hydrogels to expand and degrade makes them suitable vehicles for the encapsulation and delivery of numerous therapeutic agents, such as cells, growth factors, drugs, and proteins, to sites of tissue damage (Xu et al., 2019; Amiryaghoubi et al., 2020). These properties are ideal for sustained drug delivery applications, and CS/β-GP hydrogels are attractive biomaterials because of their temperature sensitivity. The results of rheological experiments have revealed that the gelation time and temperature could be modulated by sEVs, as they slightly decreased the gelation time and temperature. As such, after sEV loading, the resulting sEV@CS/β-GP hydrogels are attractive biomaterials for bone repair. Petit et al. reported that thermosensitive chitosan-based stain-loaded hydrogels decreased soft tissue inflammation and induced new bone formation (Petit et al., 2020), whereas Kocak et al. demonstrated that CS and hydroxyapatite composite materials loaded with low concentrations of heparin could stimulate angiogenesis and promote bone regeneration (Kocak et al., 2020).

MiRNAs regulate gene expression and biological functions by binding to the 3ʹ-UTR or amino acid coding sequence of target genes (Thomou et al., 2017). MiR-21, one of the most studied miRNAs, is involved in many biological processes (Kumarswamy et al., 2011). For example, Geng et al. reported that miR-21 can induce angiogenic differentiation of MSCs and promote blood vessel and bone formation (Geng et al., 2020). The role of SPRY2 in angiogenesis and the regulation of SPRY2 by the miR-23/27 cluster has been previously demonstrated (Zhou et al., 2011), as well as the regulation of SPRY2 by miR-21 (Thum et al., 2008). Li et al. fabricated the resultant bioswitchable nanocomposite by integrating the sticky-end tFNA (stFNA) and miRs (miR-21, miR-124, miR-335, and miR-2861), further promoting bone regeneration via inhibiting the expression of HDAC5 (Li S. et al., 2021). A similar research reported that exosomal miR-21 derived from umbilical MSC-sEVs promoted angiogenesis by upregulating the NOTCH1/DLL4 pathway (Zhang et al., 2021). In this study, the results of luciferase assays revealed that SPRY2 expression was increased by the miR-21 mimics. Furthermore, SPRY2 significantly inhibited HUVEC migration and proliferation and mediated the negative feedback of major growth factors such as VEGF, bFGF, and ANG-1. The results of gain-of-function and loss-of-function assays indicated that SPRY2 overexpression in HUVECs could attenuate, but not entirely abolish, the effects of the miR-21 mimics on angiogenesis.

## Conclusion

In summary, we developed an injectable homogeneous CS/β-GP hydrogel solution, with gelation occurring at body temperature. The hydrogel had desirable structural and physical properties that promoted bone healing, and it served as a scaffold for sEVs. The sEV-loaded hydrogel could effectively promote bone healing in a rat model by enhancing angiogenesis, which may have been mediated by the upregulation of miR-21 expression in sEVs and the regulation of SPRY2 by miR-21. This study provides a new strategy for repairing bone defects with multifunctional controlled-sEV-release hydrogels, and this sEV-based therapy shows great potential in the future.

## Data Availability

The original contributions presented in the study are included in the article/Supplementary Material, further inquiries can be directed to the corresponding authors.
