# Supplementary material for: Bone Mesenchymal Stem Cell-Derived sEV-Encapsulated Thermosensitive Hydrogels Accelerate Osteogenesis and Angiogenesis by Release of Exosomal miR-21
Source: Front Bioeng Biotechnol. 2022 Jan 19;9:829136. doi: 10.3389/fbioe.2021.829136 (PMC8807520; doi:10.3389/fbioe.2021.829136)
Supplement: Supplementary file 1 [file DataSheet1.docx]

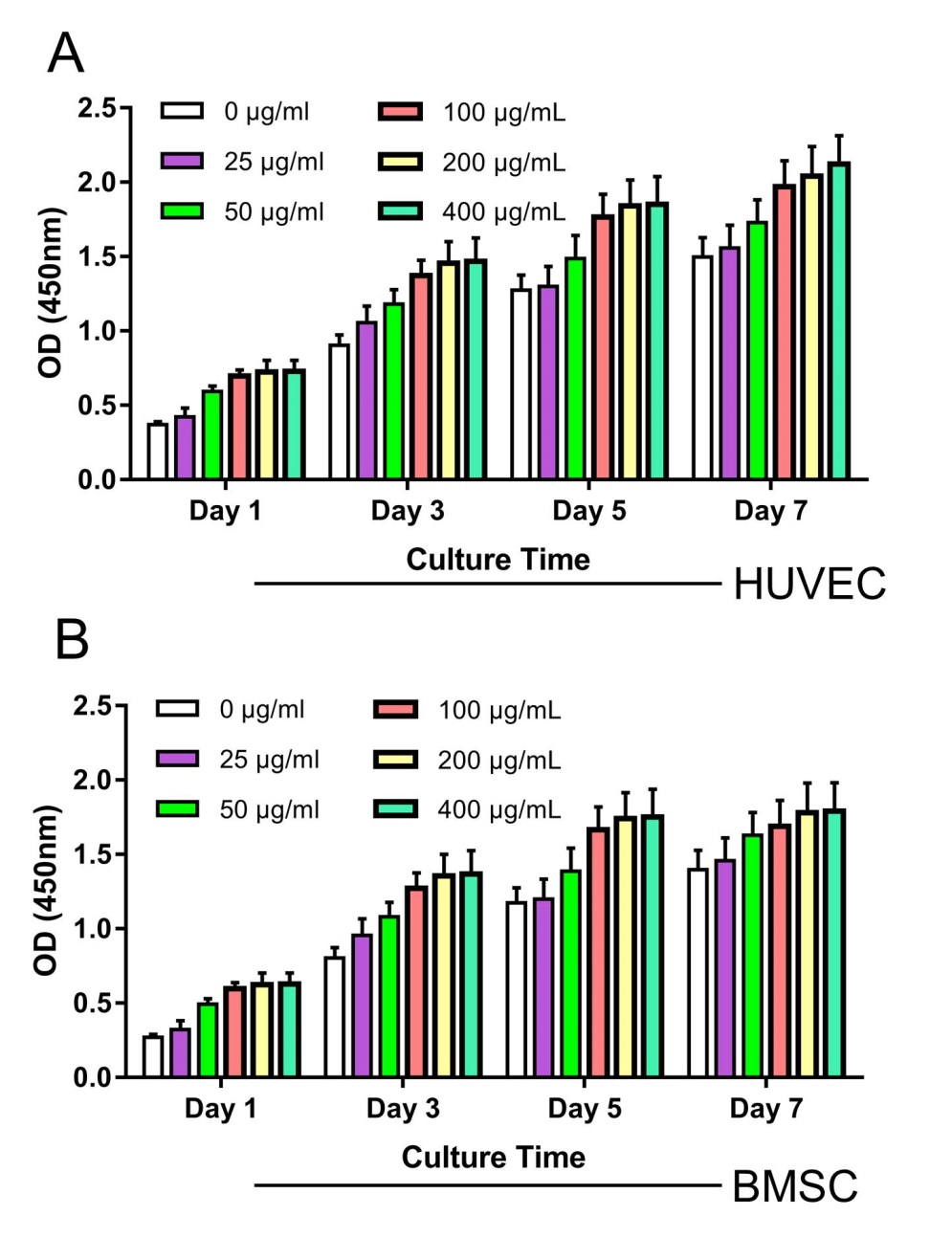


**Figure S1.** CCK8 assay for the proliferation of HUVECs (A) and BMSCs (B) co-cultured with various concentrations of sEVs.


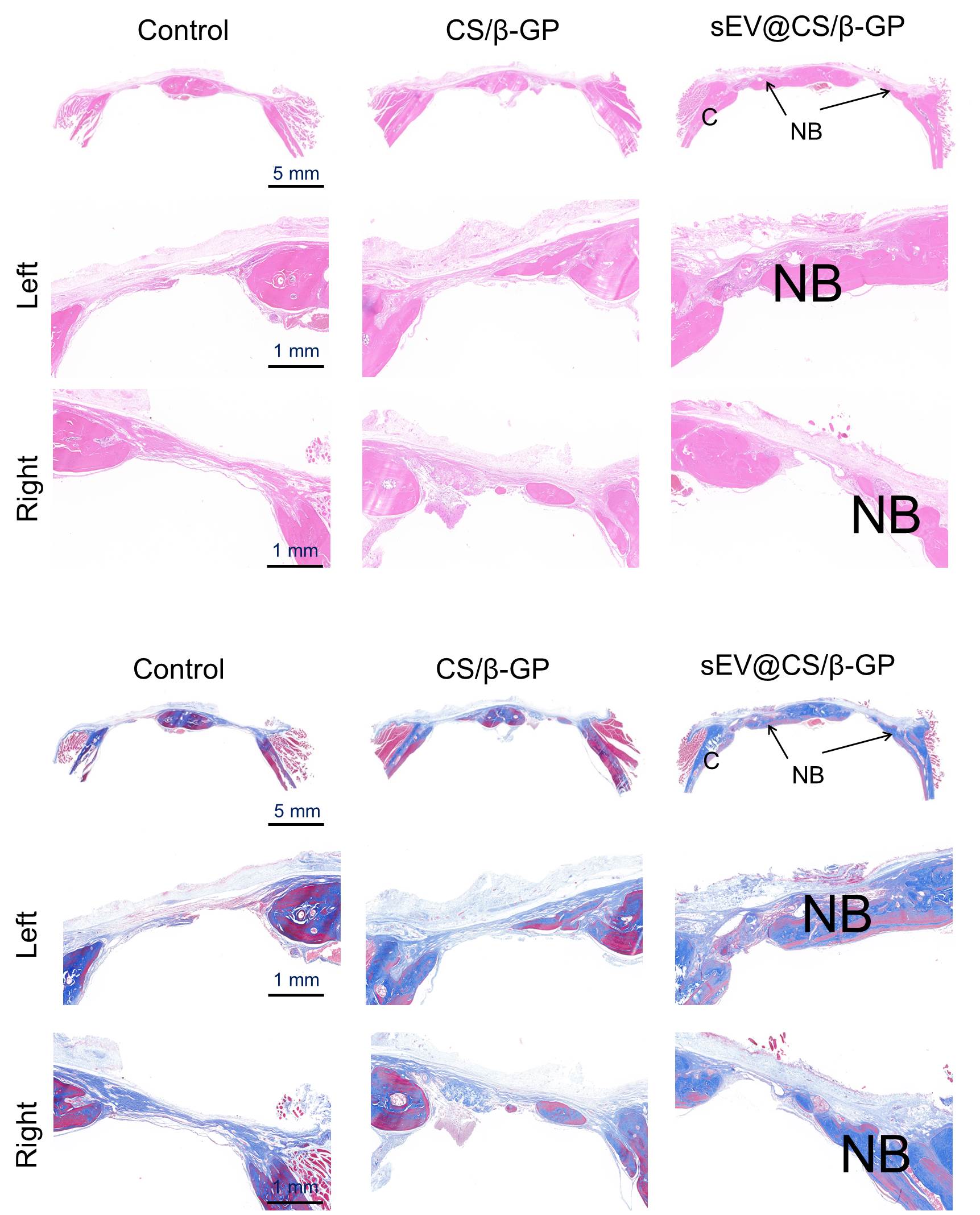


**Figure S2**. H&E staining and Masson’s trichrome staining, and magnified images of the left and right bone defects area in the three groups. C, cranium. NB, new bone.


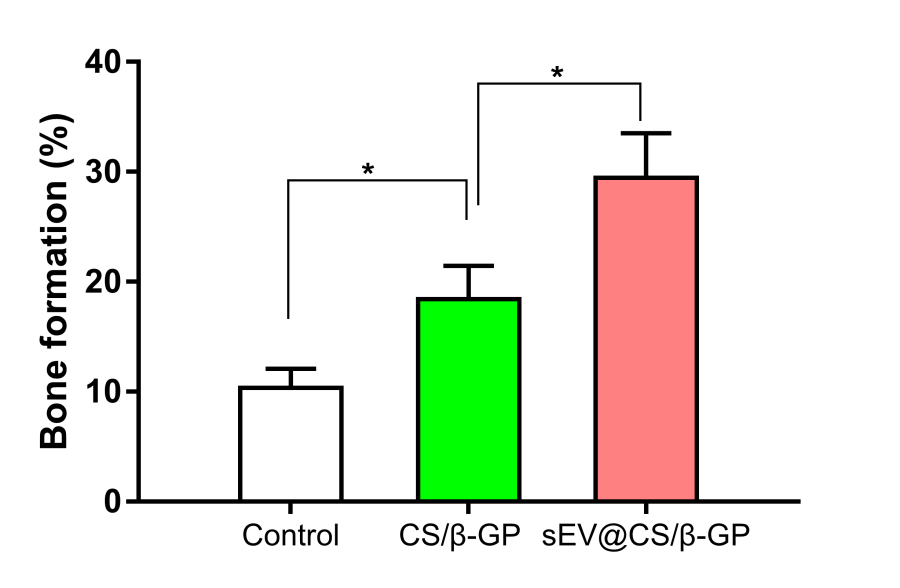


**Figure S3**. The percentage of bone tissue/newly formed bone were compared among the three groups. (*) p<0.05.
